# Morphological and Molecular Characterization of *Zanthoxylum zanthoxyloides* (Rutaceae) from Burkina Faso

**DOI:** 10.3390/plants8090353

**Published:** 2019-09-19

**Authors:** Lassané Ouédraogo, Dominik Fuchs, Hanno Schaefer, Martin Kiendrebeogo

**Affiliations:** 1 Centre National de Recherche Scientifique et Technologique (CNRST), Institut de l’Environnement et de Recherches Agricoles, 03 BP 7047 Ouagadougou 03, Burkina Faso; 2 Laboratoire de Biochimie & Chimie Appliquées, Université Joseph Ki-Zerbo, 09 BP 848 Ouagadougou 09, Burkina Faso; martinkiendrebeogo@yahoo.co.uk; 3 Plant Biodiversity Research, Technical University of Munich (TUM), Emil-Ramann Strasse 2, D-85354 Freising, Germany; dominik.fuchs@tum.de (D.F.); hanno.schaefer@tum.de (H.S.)

**Keywords:** Burkina Faso, genetic diversity, morphological characterization, *Zanthoxylum zanthoxyloides*

## Abstract

*Zanthoxylum zanthoxyloides* is a West African forest tree that is used for example against malaria and sickle cell anemia in Burkina Faso. The goal of this study was to analyze the genetic and morphological diversity of the species within wild populations in Burkina Faso, where it is potentially under threat due to the uncontrolled harvesting of its roots. Seventy-two trees from three different sites in Southwestern Burkina Faso were analyzed. Each tree was characterized by 12 traits specifying the period of flowering and maturity as well as morphological characteristics of the stem, leaves, and seeds. The molecular analysis was performed using two plastid DNA regions (*psbA-trnH* and *trnL-trnF*) and two nuclear regions (GBSSI and ITS) to identify the genetic diversity of the species for further development of a management plan for ex situ reproduction and in situ conservation. We found variability in morphological traits correlating with the geographic distance of the study sites. The molecular analysis, in contrast, revealed hardly any genetic variability among the tested trees and no population structure. Whether the differences in morphological traits are caused by different environmental conditions or by genetic variability in genes linked to morphological traits needs further testing. The apparent lack of genetic differentiation suggests that germplasm throughout the study region is suitable for planting in conservation actions. Efficient conservation management should involve local communities, especially those interested in traditional medicine.

## 1. Introduction

The genus *Zanthoxylum* (Rutaceae) evolved about 19 million years ago in Asia [1] and comprises about 250 species [2]. Recent work by Perichet et al. [3] has shown that the traditional circumscription of the genus should be revised and *Fagara* should be split from *Zanthoxylum*.

**Figure 1:**
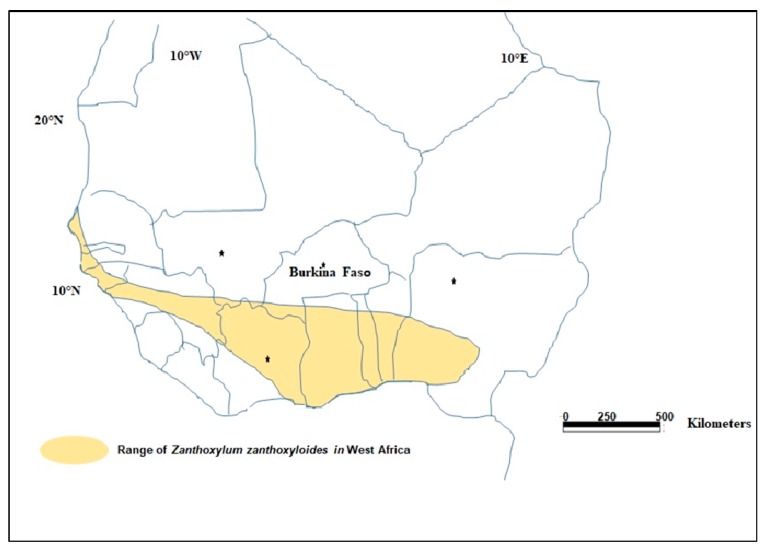
Distribution of *Zanthoxylum zanthoxyloides* in West Africa (adapted from Bonnet and Arbonnier [9]).

**Figure 2:**
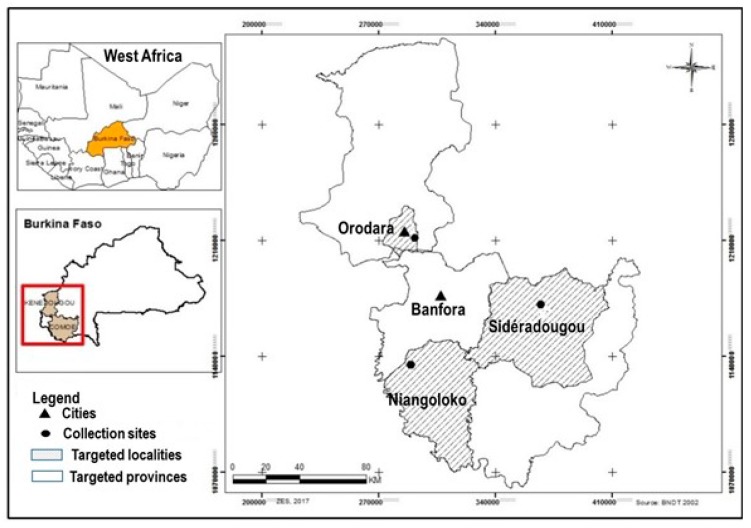
Sampling sites in the Southwest of Burkina Faso [5].

*Zanthoxylum zanthoxyloides* (Lam.) Zepernick & Timler is a small West African tree distributed from Senegal to Nigeria (Figure 1) [4]. In Burkina Faso, the plant species can be found in the southwestern part of the country (Figure 2) [5]. The plant grows as a prickly, low-branching shrub or tree, usually up to 12 meters tall, occasionally to 16 meters [6], with a diameter of up to 0.5 m [7]. In Burkina Faso, it grows in a range of habitats, including savanna, thickets, dry and transitional forests, and termite mounds [4]. The main vectors for the dispersal of this species are frugivorous animals such as migrating birds, which feed on the fruit [8].

Various parts of this species are used singly or in combination with other plants for medicinal purposes. The roots, leaves, and stem bark are commonly marketed in Cote d’Ivoire, Mali, Burkina Faso, Ghana, and Nigeria, in which they have been used in a traditional way by the rural population for their healthcare needs [10]. These plant materials serve as a remedy for the treatment of malaria, tuberculosis, ulcers, hemorrhoids, injuries, and syphilitic wounds as well as arthritic pain [10,11,12]. The roots are also used as the main ingredient to produce local phytomedicine against sickle cell anemia [10]. In Togo, hydroxy-2-methyl-benzoic acid was isolated as an active compound from this tree species and marketed in tablet-form under the name of Drepanostat^®^ for sickle cell disease treatment [13]. In Burkina Faso, FACA^®^, a phytomedicine derived from roots of *Z. zanthoxyloides* and *Calotropis procera* (Ait.) Ait.f. received the selling authorization on the market for sickle cell disease treatment [13]. Other compounds, such as Divanilloylquinic acids and Burkinabins A, B, and C, were also identified in the root bark [14,15].

About 10 species of the genus *Zanthoxylum* are already listed as endangered in the red list of IUCN [16]. Their main threats according to IUCN are the loss of their habitat, natural disasters, and deforestation. For *Z. zanthoxyloides*, the main threat is its use as a wild-collected medicinal plant [17]. In Burkina Faso, the species is limited to the southwestern corner of the country, and the species is relatively rare in its distribution area, usually reaching about five individuals per location. Since it is often the root that is used for pharmaceutical purposes, collectors tend to uproot the plants, often killing them, which results in a very unsustainable usage. An even higher future demand for this species is very likely [18] due to the increasing number of phytochemical studies highlighting its values [12,13,14]. In addition, the species is used for construction (e.g., temporary shelter for livestock [19]) and as firewood [20]. The seeds are used for seasoning, and the plant has some importance in traditional religion as protection against bad spirits [12]. Ex situ cultivation or in situ measures to enhance regeneration in nature are possible strategies to provide sufficient plant material for the phytomedicinal industry and traditional therapists and at the same time secure the long-term survival of native populations. Unfortunately, attempts to regenerate this species from seeds have not been successful so far [21], and no attempts have been made to develop a management plan for sustainable harvesting of the species.

Furthermore, the genetic diversity of wild populations of *Z. zanthoxyloides* in different regions of Burkina Faso has not yet been investigated in detail. The use and sustainable management of trees or shrubs require the analysis of genetic diversity and selection of locally adapted individuals. We therefore assessed morphological and genetic diversity of *Z. zanthoxyloides* in Burkina Faso in order to develop further cultivation or regeneration strategies with the final objective to implement conservation this useful phytogenetic resource.

## 2. Materials and Methods

### 2.1. Sites and Sampling

The sampling sites were selected on the basis of a previous study analyzing the phytochemical variability of *Z. zanthoxyloides* by spectroscopy [5]. This study had identified three sites (Niangoloko, Sidéradougou, and Orodara), where most therapists and medicine collectors gather their plant organs (see Figure 2), and these sites have also been used by Bonnet and Arbonnier [9] to sample specimens for spectral signature analyses. The site of Orodara is a wooded savanna located in the region of the Hauts-Bassins with a climate marked by a wet season between May and November and a dry season ranging from November to April. The dominant tree species in this savanna are *Vitellaria paradoxa* (C.F.Gaertn.), Sapotaceae; *Terminalia avicennioides* Guill. & Perr., *Guiera senegalensis* J.F.Gmel., and *Combretum micranthum* G.Don, Combretaceae; and *Cassia sieberiana* D.C., Fabaceae. The sites of Sidéradougou and Niangoloko are located in the forests of the Cascades region and belong to a climate of the South Sudanese type characterized by a wet season from April to October and a dry season from November to March. Climate and soil conditions of the sampling sites are summarized in Table 1. The vegetation is characterized by a mosaic of gallery forests and woody and shrub savannas [22]. The most dominant vegetation units are light forests of *Isoberlinia doka* Craib & Stapf and *Isoberlinia dalzielii* Craib & Stapf (Fabaceae) of wooded savannas with *Terminalia laxiflora* Engl. & Diels and *Terminalia mollis* M.A.Lawson (Combretaceae) and some dry forest relics with *Anogeissus leiocarpa* (DC.) Guill. & Perr. (Combretaceae) [23].

One voucher specimen per site was collected, authenticated, and stored in the herbarium of the Natural History Laboratory/National Center for Scientific and Technological Research (HNBU). These specimens were deposited under the voucher references *Ouédraogo 3061*, *3062*, and *3063*, respectively, for Niangoloko, Orodara, and Sidéradougou [5].

The study was carried out following the method described by Chehade et al. [24]. For each study site, the observations were recorded on four plots of 5000 m^2^ at a 250 m distance [5]. From each site, 24 trees of wild *Z. zanthoxyloides* of a similar growth stage were selected for morphological studies. Of each tree, 30 leaves, 30 inflorescences, and 30 seeds were sampled. This resulted in a total of 720 leaves, 720 inflorescences, and 720 seeds collected within the four plots. Among the trees used for morphological characterization, six trees per site were selected for molecular analyses, and one leaf sample was collected per tree. Soil samples were collected at a depth to 0–100 cm in each study site (Table 2), and physical parameters were analyzed (clay, silt, and sand proportion).

### 2.2. Morphological Characterization

#### 2.2.1. Quantitative and Qualitative Assessment

To compare the morphology of the trees in the three study sites, we chose a set of traits that are easy to measure without a significant impact on the tree and that had been used in previous analyses (e.g., Eyog-Matig et al. [25]; Schmelzer and Gurib-Fakim [10]). Two of the traits were qualitative traits (flowering and maturity date), and 10 were quantitative (trunk height and diameter, number of the stems, length and width of the leaf, petiole length, width and length of the inflorescence, seed diameter, and hundred-seed-weight). The phenological traits were assessed during 12 visits from December 2016 to January 2018. The quantitative traits were characterized using a tape meter, except for the seeds for which we used a vernier caliper gauge. The seeds were weighed using a balance (Adventurer Ohaus, Nänikon, Switzerland).

#### 2.2.2. Morphological Data Analysis

Analysis of variance was performed using XLSTAT 2019 to evaluate quantitative traits of the species from the three study sites. Testing for significant differences was carried out using the Newman–Keuls test at the 5% threshold. Qualitative traits were analyzed using descriptive statistical analysis (cross tabulation). A principal component analysis allowed us to assess the correlation between the morphological traits with the physical parameters of the soils from the study sites.

### 2.3. Molecular Characterization

#### 2.3.1. DNA Extraction and Quantification

Genomic DNA from dry leaf samples of *Z. zanthoxyloides* (approximately 20 mg dry leaf per sample) was extracted using the NucleoSpin Plant II DNA Kit (Machery-Nagel, Düren, Germany), in accordance with the manufacturer’s manual. The amplification of the DNA was carried out using the KAPA hifi PCR ready mix (Kapa Biosystems, Wilmington, MA, USA) according to the manufacturer’s manual. A DNA template of 20–100 ng was used in 15 µL of reaction volume. Six pairs of primers were used for amplification (Table 3). Two primer pairs amplified nuclear genes (ITS and GBSSI), and four primer pairs amplified chloroplast regions (*trnL-trnF* and *psbA-trnH*). The ITS region, *psbA-trnH*, and *trnL-trnF* are commonly used for phylogenetic studies [26,27]. The region GBSSI is a homolog of known genes in the biosynthesis of starch and important in the production of seeds [28].

#### 2.3.2. Purifying the PCR Products and Sequencing

The obtained PCR products were checked on an agarose gel and then enzymatically purified with the EXO-SAP mix (Jena Bioscience, Jena, Germany) according to the manufacturer’s protocol. Sequencing reactions consisted of 2.5 µL of clean PCR product, 5 µL of H2O, and 2.5 µL of primer (10 µM). Cycle sequencing was performed by GATC Biotech (Konstanz, Germany) with the BigDye Terminator cycle sequencing kit on an ABI Prism 3100 Avant automated sequencer (Applied Biosystems, Foster City, CA, USA).

#### 2.3.3. Editing and Alignment

Editing of the raw sequences and assembly of reads was done with Geneious R11 version 11.0.3 (Biomatters, Auckland, New Zealand). To build the alignments, we used MUSCLE [33], as implemented in Geneious. The newly generated sequences were blasted against the NCBI database ({http://www.ncbi.nlm.nih.gov/nucleotide/}) to check their identity.

## 3. Results

### 3.1. Morphological Characterization

The variance analysis indicated significant differences between the three study sites in the height of the trunk (P < 0.0001), the average diameter of the trunk (P < 0.05), and the number of stems per tree (P < 0.0001) (Table 4). The analysis of the height of the trunk showed that Sidéradougou trees (203.7 cm) were significantly higher than those of Niangoloko (151.2 cm) and the latter higher than the Orodara ones (106.5 cm). The diameter of the tree trunk from Sidéradougou (60.67 cm) was significantly larger than the ones observed for Niangoloko (45.63 cm) and Orodara (42.04 cm) trees. The average number of stems per tree ranged from 2.7 (Orodara) to 1.25 (Niangoloko) and 1.0 (Sidéradougou) (Table 4).

**Figure 3:**
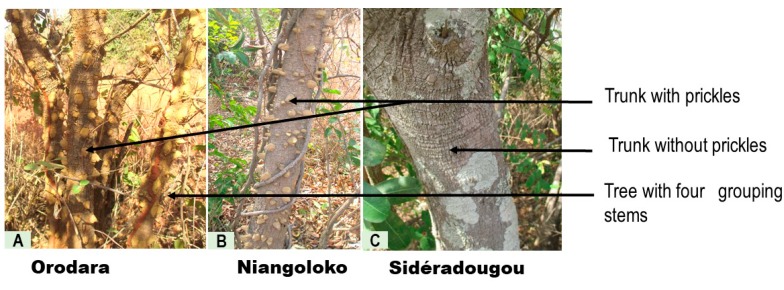
View of *Z. zanthoxyloides* stems and trunk from Orodara (**A**), Niangoloko (**B**), and Sidéradougou (**C**) evaluated in the same seasonal period.

The Sidéradougou trees are distinguished by the morphology of their single trunk, which is taller and larger than those of the other two regions and furthermore do not carry prickles. Figure 3 shows the trunk aspect of *Z. zanthoxyloides* from the three study sites.

**Figure 4:**
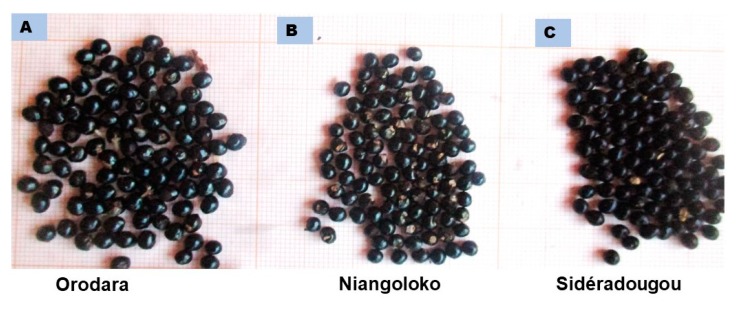
Seeds of *Z. zanthoxyloides* from Orodara (**A**), Niangoloko (**B**), and Sidéradougou (**C**).

Lengths of leaves and petioles are significantly different (P < 0.05), but there is no significant difference in the width of leaves (P = 0.891) and inflorescence (P = 0.074) between the three sampling regions (Table 5). Highly significant differences (P < 0.0001) were observed in inflorescence length, seed diameter (Figure 4), and hundred-seed weight. Inflorescence length oscillated from 5.27 (Niangoloko) to 8.83 cm (Orodara). The average diameter of the seed was in the range of 3.05 (Niangoloko) and 3.42 cm (Orodara). The hundred-seed weight varied between 2.78 (Orodara) and 2.01 g (Niangoloko). Specimens from Niangoloko are characterized by their smaller inflorescence length, seed diameter, and hundred-seed weight in comparison with those of the two other regions.

An early flowering (May–July) followed by intermediate flowering (August–October), and late flowering period (November–January) characterized *Zanthoxylum zanthoxyloides* trees. Fruit maturation also occurred in three periods: early (July–September), intermediate (October–December), and late (January–March). Most of the trees from Sidéradougou and Niangoloko flowered between August and October and the majority of the fruits matured from October to December. In contrast, most of the Orodara trees flowered and fruited in the late seasons. The flowering and fruiting periods per site are given in Table 6.

### 3.2. Correlation of Morphological Traits and Physical Parameters of Soil

There was a positive correlation (0.70 < r < 0.75) between the weight of 100 seeds, and the amount of clay (up to 30%) and silt (up to 23%). Correlation was negative (r = −0.75) between the weight of 100 seeds and sand up to 46%. The height of the trunk was positively correlated (r = 0.49) to the amount of sand up to 46%, and negatively correlated (−0.55 < r < −0.49) to clay (up to 30%) and silt up to 23%. The diameter of the trunk is negatively correlated (r = −0.26) to the amount of the silt (up to 23%). The number of stems per feet is positively correlated to the amount of clay (up to 30%) and silt up to 23% (0.67< r < 0.68) and negatively to the sand up to 46% (r = −0.67). The results of Pearson correlation are summarized in Table 7.

**Figure 5:**
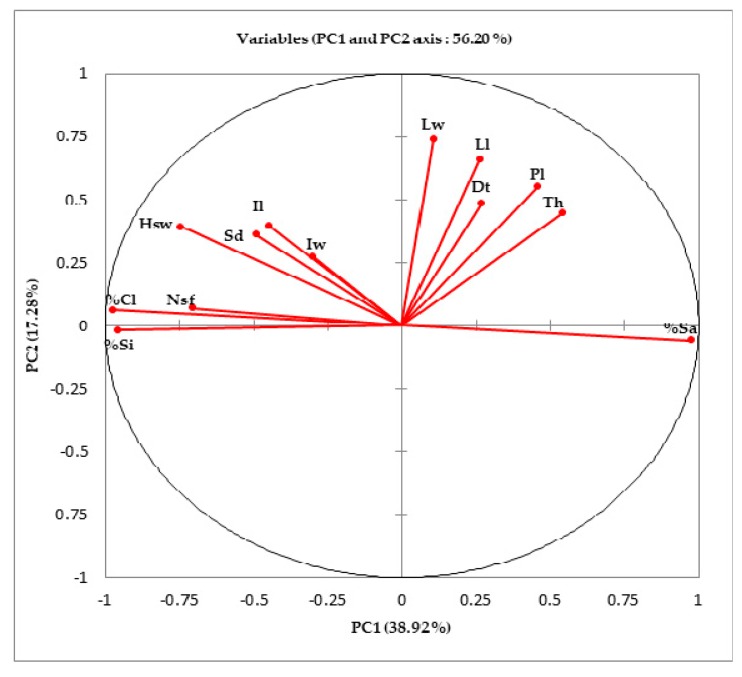
Principal Component Analysis based on plant morphology and soil physical traits.

The principal component analysis (PCA) of morphological and soils traits showed that some variables can be grouped together (Figure 5). Most variations are explained by the two first PCs with a cumulative value of 56.2% (PC1: 38.91; PC2: 17.28). The positive side of PC1 axis regrouped leaf, trunk traits, and sand percentage. The negative side of PC1 regrouped inflorescences, seeds traits, number of tree per feet, and clay and silt percentage.

### 3.3. Molecular Characterization

**Figure 6:**
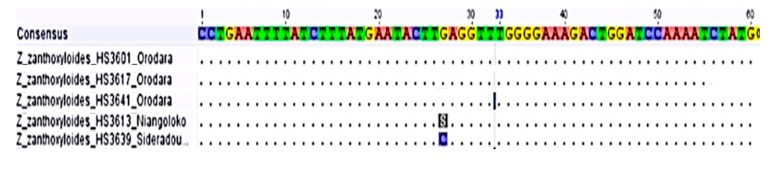
GBSSI alignment of *Z. zanthoxyloides* (partly).

The results of the PCR indicated that both the nuclear primers (ITS and GBSSI), and the chloroplast primers (*psbA-trnH* and *trnL-trnF*) exhibited good priming efficiency. In the nuclear GBSSI gene sequence (Figure 6), all the species of *Z. zanthoxyloides* were identical except for one substitution in a sample from Niangoloko and Sidéradougou, respectively.

**Figure 7:**
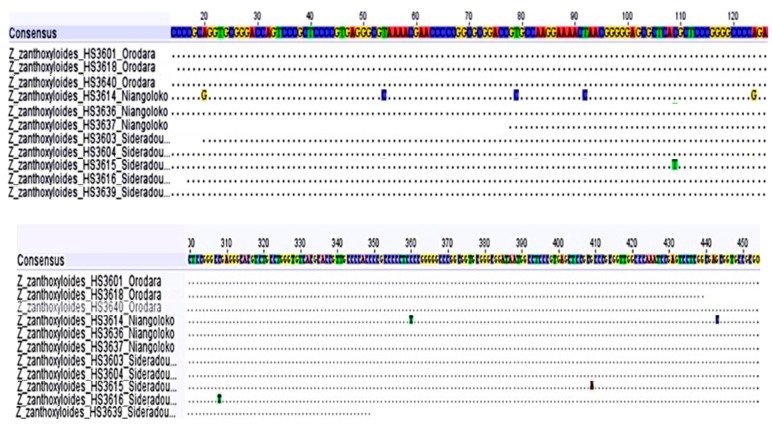
ITS alignment of *Z. zanthoxyloides* (partly).

Using the ITS primer pair, we obtained sequences from multiple samples from the three locations (n = 11). The ITS sequence alignment (Figure 7) revealed complete sequence identity in the majority of the samples (n = 8) with three exceptions. One of the three samples from Niangoloko (HS3614) exhibited seven single nucleotide exchanges more or less evenly spread along the gene. One of the five samples from Sidéradougou had two single base exchanges, and a further sample from this location showed another single nucleotide substitution. All these exchanges were autapomorphies, as they occurred only in individual samples.

**Figure 8:**
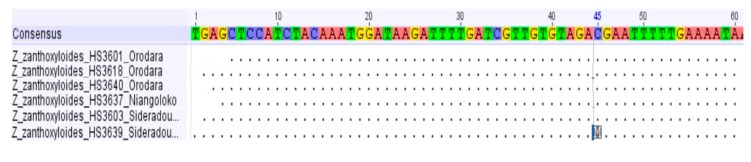
*psbA-trnH* alignment of *Z. zanthoxyloides* (partly).

In the six *psbA-trnH* sequences analyzed, we found only one exchange in one of the two samples from Sidéradougou (Figure 8).

In the two samples from Orodara and Sidéradougou, the *trnL-trnF* sequences were completely identical (data not shown).

## 4. Discussion

### 4.1. Morphological Characterization

The results of the morphological characterization indicated some variability among the sampled *Z. zanthoxyloides* populations. The height of the trunk was different in the three sites. The tallest trees were found in Sidéradougou, medium height was found in Niangoloko, and Orodara had trees of a small stunted size. Potential causes for these differences could be variations in the environmental conditions where these plants live or anthropogenic factors, such as regular cutting of the species for medicinal purposes or for collecting firewood. In fact, human action (cutting and bush fires) can affect the growth of the species, as stated by Schwartz & Caro [34]. In Orodara, we find arid clay soil, while Sidéradougou and Niangoloko have black soil with a high sand content, which could explain why the trees grow taller at the latter sites. Our results for trunk diameter are very similar, with the smallest measurements in Orodara (0.42 m), followed by Niangoloko (0.45 m), and the biggest trunks in Sidéradougou have a diameter of about 0.6 m. We can thus confirm the measurements of Eyog Matig et al. [35], who have shown that small and large forms of this species exist. The trunk diameter of 0.5 m, reported by Iwu [7], is approximately the same as what we found at Niangoloko. Another peculiarity of the Orodara trees is the increased number of stems, but it is not clear whether this is a result of frequent cutting or a natural phenomenon.

For the leaves, we determined an average size of about 17.47 cm, much larger than the 12 cm mentioned by Schmelzer and Gurib-Fakim [10]. The seed diameters from Sidéradougou and Orodora were significantly larger than the ones found in Niangoloko. In general, the seed width is in accordance with the results of Perichet et al. [3], who found a similar size for *Z. zanthoxyloides* seeds from Burkina Faso (around 3 mm). Concerning the hundred-seed weight, our results vary between 2.01 and 2.78 g, which is higher than the general weight of the species mentioned by Schmelzer and Gurib-Fakim [10] who found a weight between 1.59 and 1.81 g in the West African region. Possible explanations for these differences may be the temperature, locations, soil thickness, and forest age that would influence species development [36].

Our results from a spectroscopy study of the species indicated that the stem barks of *Z. zanthoxyloides* from Orodara have phytochemical characteristics different from Niangoloko and Sideradougou samples indicated by a closer grouping in the principal component analysis based on the fingerprint region 1800–600 cm^−1^ [5]. This region includes the absorbed O–H vibration, the C–H deformation in lignin and carbohydrates, the C–H deformation in cellulose and hemicellulose, the C–O stretch in lignin, the C–OC vibration in cellulose and hemicellulose, and the C–O stretch in cellulose and hemicellulose [37].

The multivariate analysis showed that the weight of 100 seeds correlates with the clay content of the soil (up to 30%) and silt (up to 23%). A soil with sand (up to 46%) and a proportion of clay (≤30%) and silt (≤23%) seems to have a positive effect on the height of the trunk. These results suggest that at least some of the morphological variability of *Z. zanthoxyloides* could be a consequence of the physical parameters of the soil.

### 4.2. Molecular Characterization

In the four DNA regions analyzed (GBSSI, ITS, *psbA-trnH* and *trnL-trnF*), we detected only a few nucleotide substitutions in a subset of the samples. None of these substitutions were shared by two or more trees of the same site. We conclude that these restricted substitutions are individual polymorphisms because they are not shared by two or more individuals of the same site. Thus, there are no hints that the *Z. zanthoxyloides* populations present in Burkina Faso are diverging into distinct geographic subpopulations. Of course, a numerically extended analysis spanning larger genomic regions and using microsatellites would be necessary in order to exclude any genetic diversity present in the three populations of *Z. zanthoxyloides* of our study sites. Indeed, using RAPD and ISSR primers, Mehdi et al. [38] found a significant genetic variability between various *Zanthoxylum* species and within species. Moreover, in the related *Z. giletii* from Tanzania, a clear haplotype structure could be revealed using the *psbA-trnH* intergenic spacer [39]. In populations of *Z. ailanthoides* from Kyushu Island in Japan, six haplotypes and several variations within the GBSSI gene could be detected [28]. One of the reasons why our study does not provide similar results could be the rather close vicinity of our sampling sites, which could favor an active gene flow leading to an admixture of the gene pool. Similarly, Perichet et al. [3] could not detect any molecular difference between samples of *Z. zanthoxyloides* from Burkina Faso and Ghana.

### 4.3. Conservation

So far, no adequate conservation measures have been implemented for the species in Burkina Faso. However, due to the pressure of the population using mostly the roots for medicinal purposes (malaria and sickle cell disease), the species needs to be protected and cultivated in nurseries. The results of our morphological and molecular analyses can be used to develop a conservation management plan for wild *Z. zanthoxyloides* in Burkina Faso. Our limited molecular data suggest that tree populations of *Z. zanthoxyloides* in Western Burkina have only a very low level of genetic diversity. The rare polymorphisms found do not indicate any genetic differentiation into distinct subpopulations in the three study sites. This would suggest that the variation in morphological traits among different sites is presumably due to different environmental conditions, and seeds or seedlings from all sites can probably be used for ex situ and in situ conservation measures. However, since we cannot exclude the possibility that the provenance of the seeds has an impact on seed germination or seedling growth [40], we recommend choosing young shoots or seeds from all three different provenances for conservation measures. The culture can be done in botanical gardens in pots containing soils of the different localities or clay–sand mixes. Seed can be stored in liquid nitrogen for several years [41]. To germinate the orthodox seeds, special treatment (scarifying the seeds with sand or sulfuric acid) is crucial to achieving good germination rates [40]. In addition, in vitro production by germinating the seeds in a suitable culture medium [17] can be used for small-scale production but is not an option for mass production by local communities.

Seedlings should be planted in the wet season in open areas [42] of shrub or tree savannas. To increase the acceptance and thus the survival rate of the trees, the management of these seedlings (nurseries, botanical gardens, and target places for safeguarding) should be entrusted to the local communities and especially those involved in traditional medicine, which are likely to harvest *Z. zanthoxyloides*. This should also include financial support for the very poor local communities in order to convince them to actively contribute to the conservation of the species.

## 5. Conclusions

The assessment of morphological characteristics showed diversity within the species according to different geographic sites in Southwestern Burkina Faso. However, our genetic analysis with a limited sampling did not reveal a distinct molecular diversity in the analyzed DNA regions. Whether the differences in morphological traits are caused by different environmental conditions, e.g. soil parameters, needs further testing and common-garden experiments. The conservation strategy for the species should include establishing nurseries and seed banks of *Z. zanthoxyloides* from various sites in Burkina Faso. These conservation measures should involve the local population and the actors of traditional medicine, e.g., by local management of nurseries and plantations in the selected reforestation sites.

## Figures and Tables

**Table 1:** Characteristics of the sampling sites.

Site	Geographic Coordinates (Recorded with GPS)	Altitude in m a.s.l. (Recorded with GPS)	Average Annual Precipitation from 2016–2017 (mm) (from the National Agency of Meteorology, Burkina Faso)	Average Annual Temperature 2017 (°C) from (mm) (from the National Agency of Meteorology, Burkina Faso)	Soil Type from [5]
Niangoloko	30 P 0289150 UTM 1134950	342–353	979.55	26–31	sand
Sidéra dougou	30P 0368450 UTM 1169500	320–343	771.5	26–31	sand
Orodara	30 P 0291500 UTM 1217000	546–560	980.3	26–32	clay

**Table 2:** Physical parameters of soil samples from the study sites.

Site	Depth (cm)	% Clay	% Silt	% Sand
**Niangoloko**	0–25	6.1	23.49	70.41
	25.1–50	15.2	25	59.8
	50.1–100	23.53	19.61	56.86
**Sidéradougou**	0–25	10.2	22.7	67.1
	25.1–50	16.4	25.2	58.4
	50.1–100	26.4	19.5	54.1
**Orodara**	0–25	63	25.8	11.2
	25.1–50	62.75	27.45	9.8
	50.1–100	49.6	19.61	30.79

**Table 3:** Primers used for PCR amplification.

Primers	Sequence (All Given in Orientation 5’-3’)	References
**trnLc**	CGA AAT CGG TAG ACG CTA CG	[29]
**trnLd**	GGG GAT AGA GGG ACT TGA AC	[29]
**trnLFe**	GGT TCA AGT CCC TCT ATC CC	[29]
**trnLFf**	ATT TGA ACT GGT GAC ACG AG	[29]
**ITS-F1**	CCT GCC CTT TGT ACA CAC C	[30]
**ITS-F2**	TCT CGG CAA CGG ATA TCT CG	[30]
**ITS-R1**	GCT TCT NCA GAC TAC AAT TC	[30]
**ITS-R2**	CGT TCA AAG ACT CGA TGG TTC	[30]
**psbA**	GTT ATG CAT GAA CGT AAT GCT C	[31]
**trnH**	CGC GCA TGG TGG ATT CAC AAT CC	[32]
**GBSSI f156**	GCT CCT CGC TAT GAC CAG TA	[28]
**GBSSI r642**	ACT CAA CAC CTT TAT CTT CC	[28]

**Table 4:** Tree parameters.

Site	Trunk Height (cm)	Diameter of Trunk C50	Number of Stems Per Feet
Sidéradougou (24)	203.7 ± 57.61 ^a^	60.67 ±13,40 ^a^	1.00 ± 0.000 ^b^
Niangoloko (24)	151.2 ± 50.58 ^b^	45.63 ± 27.5 ^b^	1.25 ± 0.53 ^b^
Orodara (24)	106.5 ± 50.77 ^c^	42.04 ± 21.75 ^b^	2.71 ± 1.3 ^a^
Min	27	18	1
Max	300	133	5
Mean	153.8	49.44	1.65
SqR	65.87	22.76	1.10
P-Value	<0.0001	0.009	<0.0001
Significance	HS	S	HS

The averages followed by the same lower case letter in each column are not significantly different at the 5% threshold according to the Newman–Keuls test. Min = Minimum, Max = Maximum. SqR = Standard deviation. HS = Highly significant, S = Significant. The means were calculated from 72 trees.

**Table 5:** Leaf, inflorescence, and seed parameters.

Site	Leaf Length (cm)	Leaf Width (cm)	Petiole Length (cm)	Inflorescence Length (cm)	Inflorescence Width (cm)	Seed Diameter (mm)	Hundred Seed Weight (g)
Sidéradougou (24)	18.92 ± 4.11 ^a^	11.4 ± 2.69 ^a^	4.27 ± 1.01 ^a^	8.37 ± 2.2 ^a^	2.91 ± 0.88 ^a^	3.3 ± 0.28 ^a^	2.21 ± 0.32 ^b^
Niangoloko (24)	17.02 ± 2.89 ^ab^	11.2 ± 2.39 ^a^	4.25 ± 1.15 ^a^	5.37 ± 1.68 ^b^	2.63 ± 0.51 ^a^	3.05 ± 0.07^b^	2.01 ± 0.09 ^c^
Orodara (24)	16.48 ± 2.77 ^b^	11,08 ± 1.76 ^a^	3.48 ± 0.71 ^b^	8.83 ± 2.3 ^a^	3.16 ± 0.88 ^a^	3.42 ± 0.32 ^a^	2.78 ± 0.34 ^a^
Min	10	7	2	3	2	3	1.8
Max	25	18	6	13	6	4	3.5
Mean	17.47	11.22	4	7.55	2.9	3.25	2.34
SqR	3.43	2.26	1.03	2.57	0.8	0.29	0.43
P-Value	0.03	0.891	0.008	<0.0001	0.074	<0.0001	<0.0001
Significance	S	NS	S	HS	NS	HS	HS

The averages followed by the same lower letter in each column are not significantly different at the 5% threshold after the Newman–Keuls test. Min = Minimum, Max = Maximum. SqR = Standard deviation. HS = Highly significant, S = Significant, NS = non-significant. The total number of trees for the calculation of the mean was 72.

**Table 6:** Phenology parameters.

Site	Flowering Time			Fruiting Time		
	May–July	August–October	Novemer–Jane	July–September	October–December	Jane–March
Sidéradougou	4	12	8	4	12	8
Niangoloko	9	12	3	9	12	3
Orodara	2	6	16	6	6	16
Total number of trees per season	15	30	27	15	30	27
Percentage (%)	20.83	41.67	37.5	20.83	41.67	37.5

**Table 7:** Correlation between morphological traits and physical parameters of soil.

Variables	Th	Dt	Nsf	Lf	Lw	Pl	Hsw	Sd	Il	Iw	%Cl	%Si	Sa
Th	**1**												
Dt	**0.3107**	**1**											
Nsf	**−0.2741**	**−0.2396**	**1**										
Ll	**0.2419**	0.1755	−0.0565	**1**									
Lw	0.1449	**0.2412**	0.0370	**0.6249**	**1**								
Pl	**0.5091**	0.2148	−0.1982	**0.3706**	**0.4132**	**1**							
Hsw	−0.1665	−0.0405	**0.4180**	0.0388	0.1620	−0.0838	**1**						
Sd	0.0430	−0.0045	**0.3004**	0.0023	0.0382	−0.1016	**0.4733**	**1**					
Il	0.0082	0.1938	0.1903	−0.0373	0.0203	−0.1489	**0.4015**	**0.4079**	**1**				
Iw	−0.0919	0.1267	0.1041	−0.0564	0.0642	−0.0684	0.1733	0.1659	**0.4707**	**1**			
%Cl	**−0.4925**	−0.2164	**0.6756**	−0.1931	−0.0416	**−0.3596**	**0.7539**	**0.4298**	**0.3880**	**0.2419**	**1**		
%Si	**−0.5541**	**−0.2694**	**0.6880**	**−0.2372**	−0.0488	**−0.3544**	**0.7073**	**0.3541**	**0.2869**	0.2096	**0.9793**	**1**	
%Sa	**0.4943**	0.2179	**−0.6762**	0.1943	0.0418	**0.3596**	**−0.7531**	**−0.4281**	**−0.3856**	**−0.2412**	**−1.0000**	**−0.9804**	**1**

Values in bold are significant for P < 0,05. Th: Trunk height, Dt: Diameter of trunk, Nsf: Number of stems per feet, Ll: Leaf length, Lw: Leaf width, Pl: Petiole length, Hsw: Hundred seed weight, Sd: Seed diameter, Il: Inflorescence length, Iw: Inflorescence width, %: Proportion in percentage, Cl: Clay, Si: Silt, Sa: Sand.
